# Impact of Chemical Analogs of 4-Hydroxybenzoic Acid on Coenzyme Q Biosynthesis: From Inhibition to Bypass of Coenzyme Q Deficiency

**DOI:** 10.3389/fphys.2017.00436

**Published:** 2017-06-22

**Authors:** Fabien Pierrel

**Affiliations:** Centre National de la Recherche Scientifique, Grenoble INP, TIMC-IMAG, University Grenoble AlpesGrenoble, France

**Keywords:** coenzyme Q, CoQ deficiency, mitochondrial disease, 4-hydroxybenzoic acid, para-aminobenzoic acid, biosynthesis, chemical analogs, bioavailability

## Abstract

Coenzyme Q is a lipid that participates to important physiological functions. Coenzyme Q is synthesized in multiple steps from the precursor 4-hydroxybenzoic acid. Mutations in enzymes that participate to coenzyme Q biosynthesis result in primary coenzyme Q deficiency, a type of mitochondrial disease. Coenzyme Q_10_ supplementation of patients is the classical treatment but it shows limited efficacy in some cases. The molecular understanding of the coenzyme Q biosynthetic pathway allowed the design of experiments to bypass deficient biosynthetic steps with analogs of 4-hydroxybenzoic acid. These molecules provide the defective chemical group and can reactivate endogenous coenzyme Q biosynthesis as demonstrated recently in yeast, mammalian cell cultures, and mouse models of primary coenzyme Q deficiency. This mini review presents how the chemical properties of various analogs of 4-hydroxybenzoic acid dictate the effect of the molecules on CoQ biosynthesis and how the reactivation of endogenous coenzyme Q biosynthesis may achieve better results than exogenous CoQ_10_ supplementation.

## Introduction

**Figure 1:**
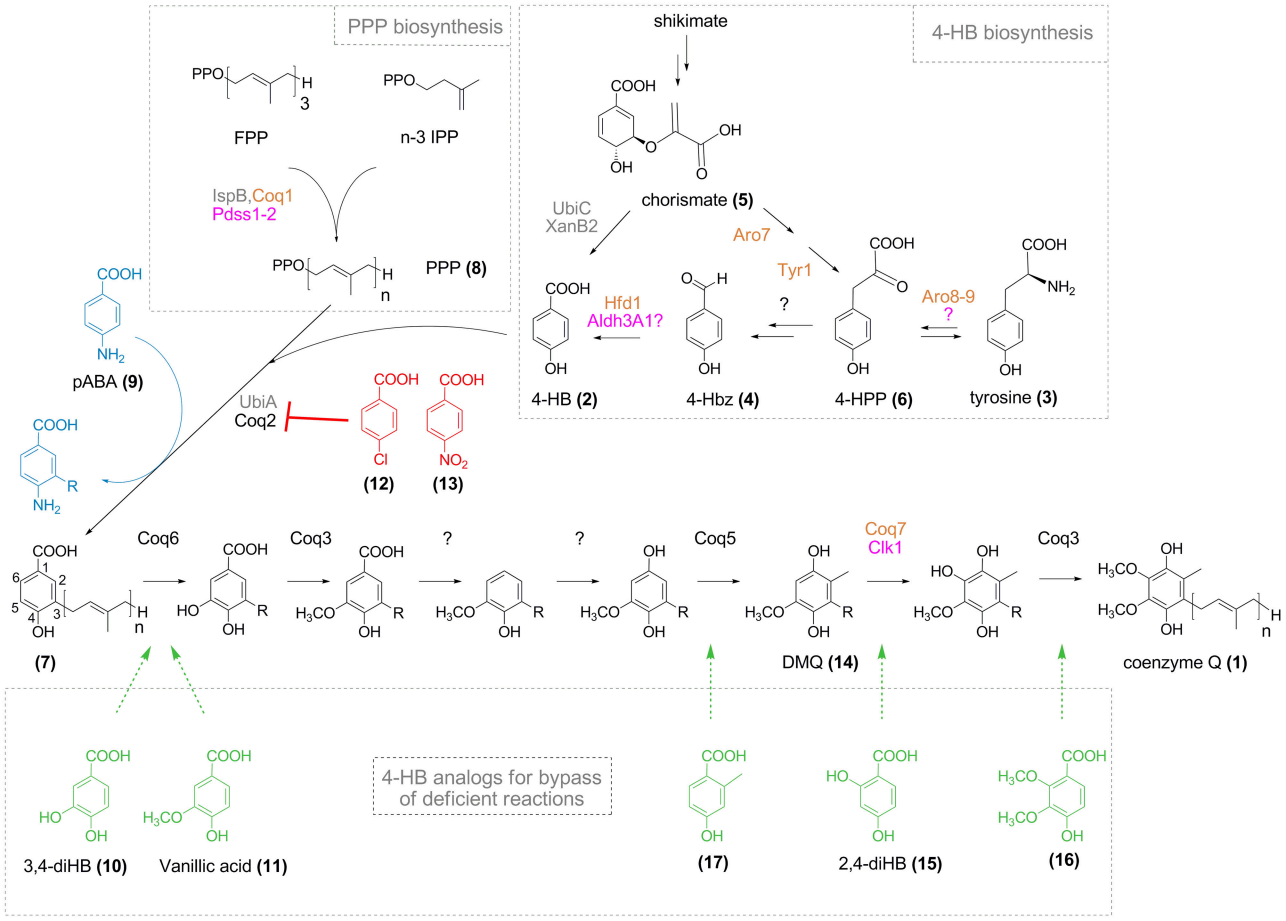
CoQ biosynthesis and effect of 4-HB analogs. Names for enzymes of *E. coli, S. cerevisiae*, and humans are in gray, orange, and pink, respectively (in black when common to *S. cerevisiae* and humans). Polyprenyl pyrophosphate (PPP, **8**, *n* = 6–10) synthesized from farnesyl pyrophosphate (FPP) and n-3 isopentenyl pyrophosphate (IPP) is conjugated to 4-HB (**2**) by Coq2/UbiA to yield polyprenyl-4-HB (**7**). The numbering of the aromatic carbon atoms is shown on polyprenyl-4-HB and is uniformly applied to the different intermediates cited in the text, although IUPAC nomenclature might be different depending on the substitution pattern. Since the order of some reactions is different in bacteria (Aussel et al., 2014), only the eukaryotic pathway from polyprenyl-4-HB to CoQ is shown. R corresponds to the polyprenyl moiety (*n* = 6–10). 4-Chlorobenzoic acid (**12**) and 4-nitrobenzoic acid (**13**) (red) are inhibitors of Coq2/UbiA, whereas pABA (**9**, blue) is prenylated and progress to different stages of the CoQ biosynthetic pathway depending on the organisms (see text for details). 4-HB analogs (in green) have potential (**16, 17)** or proven (**10, 11, 15**) capacities to bypass the biosynthetic steps indicated by the dashed arrows.

Coenzyme Q (CoQ, compound **1** on Figure 1), also known as ubiquinone, is a lipid conserved from proteobacteria to humans. CoQ is composed of a benzoquinone ring that is attached to a polyisoprenyl tail of various length (six isoprenyl units in *Saccharomyces cerevisiae* hence CoQ_6_, ten units in humans, hence CoQ_10_). The benzoquinone ring is redox active and exchanges two electrons and two protons between the oxidized and reduced forms of CoQ, which play numerous roles in cellular physiology (Bentinger et al., 2010; Wang and Hekimi, 2016).

In eukaryotic cells, the biosynthesis of CoQ takes place at the mitochondrial inner membrane (Wang and Hekimi, 2013a) and also possibly in the Golgi apparatus (Mugoni et al., 2013). 4-hydroxybenzoic acid (4-HB, **2**) is the precursor of the benzoquinone ring of CoQ. 4-HB is first prenylated by Coq2 (UbiA in bacteria) and then, a total of seven reactions—one decarboxylation, three hydroxylation, and three methylation—produce the fully substituted benzoquinone ring of CoQ (Figure 1). Even though the structure of CoQ was established almost 60 years ago (Lester et al., 1958; Morton, 1958), the identity of the enzymes that catalyze the decarboxylation reaction and one of the three hydroxylation reaction is still elusive in eukaryotes (Kawamukai, 2016). In addition, the pathway that converts tyrosine **(3)** into 4-HB is poorly characterized and the last reaction, the oxidation of 4-hydroxybenzaldehyde (4-Hbz, **4**) to 4-HB was only recently elucidated in *S. cerevisiae* (Payet et al., 2016; Stefely et al., 2016).

Primary CoQ_10_ deficiency, caused by mutations in genes involved in CoQ biosynthesis, is a rare condition with a heterogeneous clinical spectrum. Mutations in *PDSS1, PDSS2, COQ2, COQ4, COQ6, COQ7, COQ9, ADCK3*, and *ADCK4* have been identified to date (Acosta et al., 2016). CoQ deficiency represents one of the few mitochondrial disorder that is treatable (Hirano et al., 2012), although not all patients respond to oral CoQ_10_ supplementation (Hirano et al., 2012). The success of the treatment is influenced by the advance of the disease at the time when CoQ_10_ supplementation is initiated (Acosta et al., 2016). The poor bioavailability of CoQ_10_ also contributes to the variable results of CoQ_10_ supplementation. Indeed, the lipophilic nature of CoQ_10_ is thought to limit its distribution in the human body and its transport to the mitochondrial inner-membrane (Bentinger et al., 2003; Lopez et al., 2010; Hirano et al., 2012).

This mini-review will discuss how various 4-HB analogs impact CoQ biosynthesis and how some of them bypass altered biosynthetic steps, as first demonstrated in *S. cerevisiae* by my group and that of Catherine Clarke (Ozeir et al., 2011; Xie et al., 2012). I will also present the potential benefits and limitations of using analogs of 4-HB over CoQ_10_ supplementation to treat CoQ_10_ deficiency linked to mutations in specific genes.

## Biosynthesis of 4-HB, the natural precursor of the aromatic ring of CoQ

4-HB together with 4-Hbz have been suspected early on as potential precursors of the benzoquinone ring of CoQ in animals, yeast, and bacteria (Parson and Rudney, 1964). In *Escherichia coli*, 4-HB is produced by a chorismate pyruvate-lyase reaction catalyzed by UbiC (Nichols and Green, 1992; Siebert et al., 1992), which substrate is chorismic acid **(5)**, an intermediate of the shikimate pathway that feeds the biosynthesis of aromatic amino acids (Lawrence et al., 1974). However, many proteobacteria that synthesize CoQ lack an *ubiC* homolog and the widespread *xanB2* gene was recently shown to encode a bifunctional enzyme that converts chorismate into either 4-HB or 3-HB (Zhou et al., 2013). Animals do not possess the shikimate pathway and derive 4-HB from tyrosine and phenylalanine (Olson et al., 1965; Olson, 1966), via a pathway that remains putative (Clarke, 2000), but potentially implicates para-coumarate (Xie et al., 2015). In the plant *Arabidopsis thaliana*, phenylalanine and tyrosine independently contribute to the synthesis of 4-HB (Block et al., 2014). Only the pathway originating from phenylalanine has been partially characterized and involves β-oxidation in peroxisomes (Block et al., 2014).

Unlike bacteria, *S. cerevisiae* does not produce 4-HB in a single step from chorismate. Instead, pathways from shikimate or exogenous tyrosine converge at 4-hydroxyphenyl pyruvate (4-HPP, **6**), which is further converted to 4-Hbz via uncharacterized steps (Payet et al., 2016) (Figure 1). As a final reaction, 4-Hbz is oxidized to 4-HB by the aldehyde dehydrogenase Hfd1 (Payet et al., 2016; Stefely et al., 2016). Since the human homolog *ALDH3A1* complements the defects of yeast Δ*hfd1* cells for CoQ biosynthesis and respiratory growth (Payet et al., 2016; Stefely et al., 2016), the oxidation of 4-Hbz to 4-HB may also take place in humans, as suspected from early results in animals (Parson and Rudney, 1964). The elucidation of the human pathway from tyrosine to 4-HB is important as mutations in participating genes may result in CoQ deficiency, which may be compensated by supplying 4-HB. Indeed, exogenous 4-HB rescues the levels of CoQ in mutants that disrupt 4-HB biosynthesis in bacteria, yeast, and plants (Zhou et al., 2013; Block et al., 2014; Payet et al., 2016; Stefely et al., 2016).

## Rules for the prenylation of 4-HB analogs

4-HB enters the CoQ biosynthetic pathway via the prenylation of the position 3 catalyzed by Coq2 in eukaryotes and UbiA in bacteria that yield 3-polyprenyl-4-hydroxybenzoic acid **(7)**. The polyprenyl pyrophosphate (PPP, **8**) is formed by Coq1/Pdss1-Pdss2 (Kawamukai, 2016) (Figure 1). Aromatic compounds are substrates of UbiA if their carbon C3 is activated by a carboxylic acid moiety on C1 and a group on C4 that is electron- and hydrogen bond-donor, like hydroxyl or amine groups (Wessjohann and Sontag, 1996). Electron-withdrawing groups on C4, such as nitro and chloro, led to loss of activity of UbiA (Wessjohann and Sontag, 1996; Brandt et al., 2009). Interestingly, substituents in position 5 and 6 of 4-HB are tolerated.

These structural requirements are also applicable with the rat Coq2 enzyme since para-aminobenzoic acid (pABA, **9**), 3,4-dihydroxybenzoic acid (3,4-diHB, **10**), and 4-hydroxy-3-methoxybenzoic acid (vanillic acid, **11**) were prenylated in cell free extracts, whereas chlorobenzoic acid **(12)** inhibited the prenyl transferase reaction (Alam et al., 1975; Nambudiri et al., 1977). Furthermore, 4-nitrobenzoic acid (4-NB, **13**) was shown to be a competitive inhibitor of Coq2 and to decrease CoQ biosynthesis in mammalian cell cultures in a dose- and time-dependent manner (Forsman et al., 2010). 4-NB was subsequently used to evaluate how different residual levels of CoQ impact various cellular parameters (Quinzii et al., 2012), contributing to a better understanding of the pathomechanisms underlying primary CoQ deficiency. Altogether, these studies demonstrate that the active site of Coq2-UbiA can accommodate several 4-HB analogs which will act as substrates or inhibitors depending on their chemical properties.

The crystal structures of two UbiA homologs from archaeal thermophiles have recently been reported (Cheng and Li, 2014; Huang et al., 2014). Both membrane-embedded proteins present nine transmembrane helices. In one structure, a lateral portal delineated by two transmembrane domains was proposed to open to the membrane and allow access to the PPP molecule (Cheng and Li, 2014). Two conserved aspartate-rich motifs are located in a central cavity and coordinate two Mg^2+^ ions involved in binding the PPP analogs used in co-crystallization experiments. Unfortunately, no details are available regarding the coordination of 4-HB in the active site. Indeed, in one enzyme, 4-HB binding could not be detected by isothermal titration calorimetry and tentative 4-HB modeling clashed with the position occupied by the PPP analog (Huang et al., 2014) whereas in the other enzyme, 4-HB was modeled so that its carboxyl group contacts Arg43 (Cheng and Li, 2014). Yet, the mutation of the corresponding arginine residue in *E. coli* UbiA did not completely abolish activity (Cheng and Li, 2014), raising doubts about the involvement of this residue in the coordination of 4-HB.

## pABA advances to different stages of CoQ biosynthesis depending on the organisms

pABA fulfills the requirements for prenylation by the Coq2-UbiA prenyltransferases and labeling experiments demonstrated that pABA is converted to CoQ in *S. cerevisiae* (Marbois et al., 2010; Pierrel et al., 2010). Consequently, the C4 amino group of pABA must be replaced by a C4 hydroxyl group and we recently reported that the monooxygenase Coq6 catalyzes this reaction in addition to the previously reported C5-hydroxylation (Ozeir et al., 2015). The L382E mutation in Coq6 severely impairs the C4-deamination reaction but globally maintains the C5-hydroxylation (Ozeir et al., 2015). Δ*coq9* cells accumulate C4-aminated intermediates of the CoQ pathway (Xie et al., 2012; He et al., 2015) but Coq9 plays an indirect role in the C4-deamination as its absence impacts the C-terminal region of Coq6, which is important for the C4-deamination but quite dispensable for the C5-hydroxylation (Ozeir et al., 2015).

**Figure 2:**
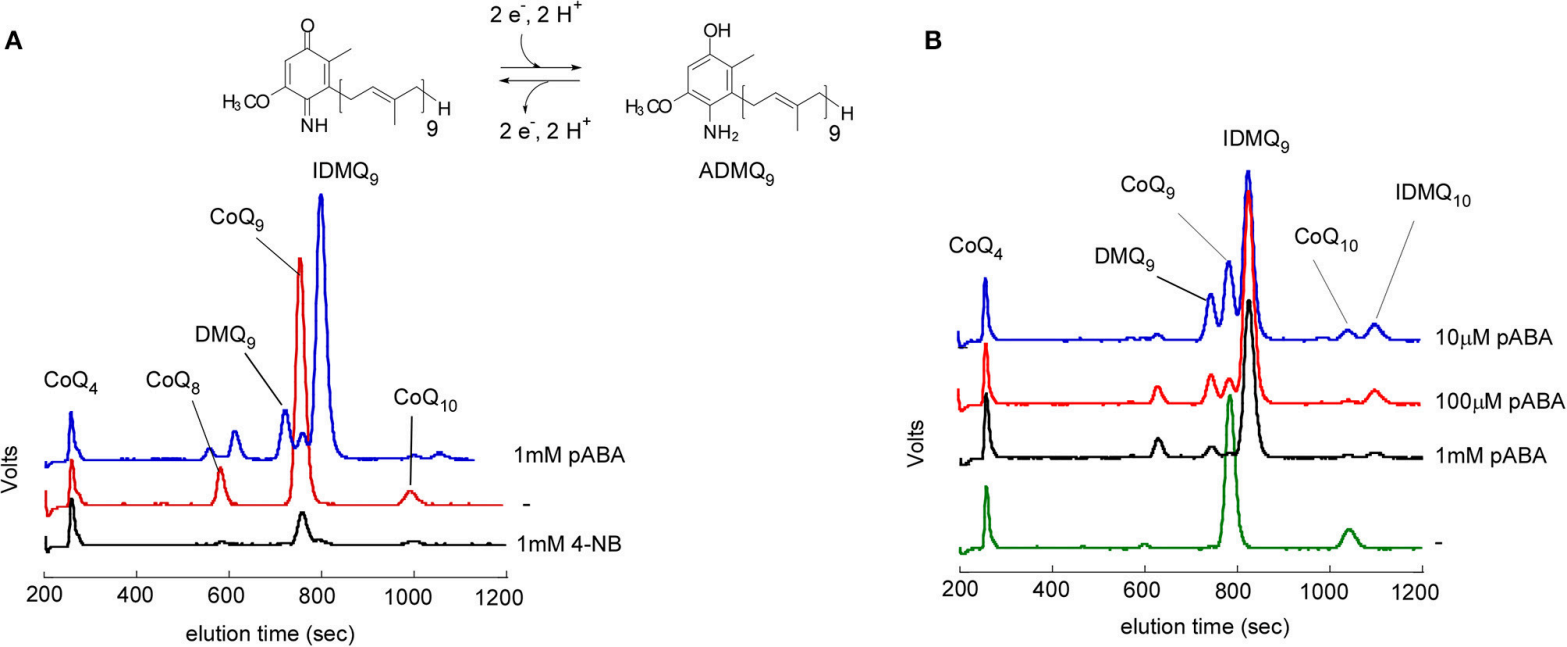
Effect of pABA on mammalian cell lines. Analysis of cellular lipid extracts by high performance liquid chromatography coupled to electrochemical detection with a precolumn electrode set in oxidizing mode. NIH/3T3 fibroblasts **(A)** and CHO cells **(B)** were grown for one week in the absence (−) or with the indicated concentrations of pABA or 4-NB. CoQ_4_ was used as an internal standard. The peaks corresponding to CoQ_9_ and CoQ_10_, 4-imino-6-demethoxyubiquinone 9 (IDMQ_9_) and IDMQ_10_, 6-demethoxyubiquinone 9 (DMQ_9_) are indicated. The chemical structure of IDMQ_9_ and ADMQ_9_ are shown.

So far, pABA was reported to be a precursor of CoQ only in *S. cerevisiae*. *E. coli*, the plant *Arabidopsis*, or mammalian cells do not incorporate pABA into CoQ (Block et al., 2014; Xie et al., 2015). In *E. coli*, prenylated pABA is decarboxylated and hydroxylated to yield 2-amino-3-octaprenylphenol, which was suggested to be a “dead-end” product (Xie et al., 2015). In mammalian cell cultures, pABA decreases CoQ levels (Gonzalez-Aragon et al., 2005; Xie et al., 2015) and unidentified prenylated compounds were previously detected (Alam et al., 1975; Forsman et al., 2010). Indeed, results from my group confirmed a strong decrease of cellular CoQ_9_ in Chinese Hamster Ovary (CHO) cells and NIH/3T3 fibroblasts (derived from Swiss mouse embryo tissue) treated with pABA (Figure 2). In addition, both cell lines accumulate a major redox compound, which was identified as 4-imino-6-demethoxycoenzyme Q_9_ (IDMQ_9_) by high resolution mass spectrometry. IDMQ_9_ can be reduced to 4-amino-6-demethoxycoenzyme Q_9_ (ADMQ_9_) (Figure 2), similarly to IDMQ_6_ that was previously detected in yeast Δ*coq9* cells (Xie et al., 2012). These results suggest that the C4-deamination is not taking place in mammalian cells and that Clk1-Coq7 cannot hydroxylate IDMQ or ADMQ (I-ADMQ). The properties of I-ADMQ are not known but they may have antioxidant capacities and may also participate to mitochondrial respiration as demonstrated for the closely related molecule demethoxy-coenzyme Q (DMQ, **14**; Wang and Hekimi, 2013b). Therefore, pABA may not be the most appropriate agent to induce CoQ_10_ deficiency in cell lines and study the consequential physiological impacts (Gonzalez-Aragon et al., 2005; Duberley et al., 2013, 2014). Instead, 4-NB must be considered for such purposes since it does not form any prenylated products (Forsman et al., 2010; Quinzii et al., 2012).

Mammalian cells do not synthesize pABA contrary to microorganisms, which derive pABA from the shikimate pathway (Botet et al., 2007). pABA is frequently found in nutritional supplements but is rapidly and efficiently eliminated from the human body (Sharma et al., 2014), limiting the risk of interference with CoQ biosynthesis. Accordingly, pABA did not perturb CoQ biosynthesis in mice. Indeed the levels of CoQ_9_ were maintained in various tissues—heart, brain, lung, spleen, kidney, liver, and skeletal muscle—of C57BL/6 mice injected intraperitoneally with pABA (50 mg/kg/day) for 4 weeks and I-ADMQ_9_ were not detected (unpublished results). Thus, pABA seems to interfere with mammalian CoQ biosynthesis only in cell cultures.

## Using 4-HB analogs to bypass deficient steps in CoQ biosynthesis

The possibility to bypass a deficient step in CoQ biosynthesis by providing the defective chemical group within a synthetic analog of 4-HB was first demonstrated with 3,4-diHB and vanillic acid (Ozeir et al., 2011). Both compounds restored CoQ biosynthesis in *S. cerevisiae* cells impaired in C5-hydroxylation because of mutations in *coq6* (Ozeir et al., 2011; Doimo et al., 2014). When working with knock-out *S. cerevisiae* mutants, the effect of 4-HB analogs is dependent upon overexpression of Coq8 to stabilize several Coq proteins that are instable in Δ*coq* strains (Padilla et al., 2009; Xie et al., 2012; He et al., 2014). 2,4-Dihydroxybenzoic acid (2,4-diHB, **15**) bypassed a C6-hydroxylation defect and allowed CoQ6 biosynthesis in Δ*coq7* yeast cells overexpressing *Coq8* (Xie et al., 2012). 2,4-diHB increased CoQ_10_ levels in fibroblasts with an homozygous V141E mutation in *COQ7* (Freyer et al., 2015) but was inefficient with the L111P mutation (Wang et al., 2017).

Besides cell cultures, 2,4-diHB was also efficient in mice with an inducible deletion of Coq7 (also called Mclk1). Addition of 2,4-diHB to the drinking water shortly after induction of the Mclk1 deletion increased CoQ_9_ levels in heart, kidney, and skeletal muscle and markedly rescued the mutant phenotypes, including mitochondrial respiration, blood lactate levels, and lifespan (Wang et al., 2015). In this model, dietary CoQ_10_ supplementation was almost without an effect, likely because of poor tissue uptake except by the liver (Wang et al., 2015). Interestingly, 2,4-diHB was also efficient when provided as a late treatment, i.e., when the symptoms had already developed. Oral 2,4-diHB also proved efficient in the Coq9^R239X^ mouse, as it increased the kidney CoQ_9_ content (Luna-Sanchez et al., 2015). Coq9^R239X^ mice have decreased CoQ_9_ and accumulate DMQ_9_ (Garcia-Corzo et al., 2013) as the Coq7 reaction is impeded since Coq9 interacts with Coq7 and is thought to facilitate its function (Lohman et al., 2014). Together, these results suggest that 2,4-diHB may be beneficial to patients with primary CoQ deficiency due to *COQ7* or *COQ9* mutations.

Such bypass strategies may be applicable to other cases of primary coenzyme Q deficiency. *COQ6* patients (Heeringa et al., 2011) may respond to vanillic acid or 3,4-diHB (Doimo et al., 2014). Although disease-causing mutations in *COQ3* and *COQ5* genes have not been reported yet, 2,3-dimethoxy-4-hydroxybenzoic acid **(16)** and 2-methyl-4-hydroxybenzoic acid **(17)** may, in theory, bypass the requirement for Coq3 and Coq5, respectively. The possibility for the former compound to be prenylated by Coq2 is supported by the *in vitro* prenylation of the related 2,3,4-trihydroxybenzoic acid (2,3,4-tri-HB) by UbiA (Wessjohann and Sontag, 1996). Altogether, several reactions of the CoQ biosynthetic pathway are potentially amenable to bypass. However, it is impossible to circumvent the C1 decarboxylation or C1 hydroxylation reactions with analogs bearing a hydroxyl group on C1, since the presence of a carboxyl group at C1 of the benzene ring is indispensable for prenylation (Alam et al., 1975; Wessjohann and Sontag, 1996).

## Advantages of using analogs of 4-HB that restore endogenous CoQ biosynthesis over exogenous CoQ_10_ supplementation

To replenish CoQ levels in CoQ deficient cells and organisms, the use of bypass 4-HB analogs may be advantageous over CoQ_10_ supplementation for the following reasons. (i) 4-HB analogs will allow to preserve the endogenous ratio between the major and minor isoforms of CoQ. Indeed, many species have a prominent CoQ isoform (CoQ_9_ in rodents, CoQ_10_ in humans) but also synthesize minor isoforms (CoQ_10_ in rodents, CoQ_9_ in humans). The ratio of both isoforms varies significantly depending on the organs (Turunen et al., 2004), yet, it remains unknown whether these varying ratios have any physiological consequences. (ii) Thanks to their hydrophilic nature, 4-HB analogs may have a superior bioavailability than exogenously supplied CoQ_10_, which accumulates efficiently in the liver but not in other organs (Miles, 2007). Indeed, CoQ_10_ supplementation of CoQ deficient mouse models did not increase CoQ_10_ content in kidney (Saiki et al., 2008) or heart or muscle (Wang et al., 2015). However, a new formulation of CoQ_10_ demonstrated improved bioavailability as it increased CoQ_10_ levels in all tested organs of Coq9^R239X^ mice, although to a limited extent for most of them (Garcia-Corzo et al., 2014). (iii) 4-HB analogs may achieve higher CoQ levels in organs than CoQ_10_ supplementation. 2,4-diHB doubled the kidney CoQ_9_ content of Coq9^R239X^ mice (Luna-Sanchez et al., 2015) and tripled that of Mclk1-deficient mice (Wang et al., 2015), although WT levels were not reached in either model. For comparison, CoQ_10_ supplementation yielded a ~50% increase of total kidney CoQ (CoQ_9_+CoQ_10_) in the former model (Garcia-Corzo et al., 2014) and none in the latter (Wang et al., 2015). (iv) CoQ produced from 4-HB analogs should distribute normally between subcellular compartments whereas exogenously supplied CoQ_10_ has difficulties to reach mitochondria and their inner membrane (Bentinger et al., 2003). (v) Short chains analogs of CoQ like idebenone and decylubiquinone have been reported to increase superoxide production (Genova et al., 2003), but 4-HB analogs are not expected to have such effect since they don't have a redox-active benzoquinone moiety.

## Potential limitations to the use of 4-HB analogs

The successful restoration of endogenous CoQ biosynthesis by 4-HB analogs depends on several factors. (i) The 4-HB analogs must outcompete endogenous 4-HB in the prenylation reaction catalyzed by Coq2. Thus, the Km of Coq2 for the analog should be in the same range as that for 4-HB or the analog should be substantially more abundant than 4-HB. (ii) Except for the defective CoQ biosynthetic step, all other enzymatic reactions must be maintained. However, many CoQ biosynthetic proteins form a complex in human cells (Floyd et al., 2016) and the abundance of several Coq proteins decreased in multiple tissues of the Coq9^R239X^ mice (Lohman et al., 2014; Luna-Sanchez et al., 2015), reflecting the instability of an incompletely assembled complex, as already observed in yeast (Hsieh et al., 2007; Xie et al., 2012). (iii) The Coq enzymes must be able to modify unnatural substrates with extra chemical groups. For example, Coq6, which usually hydroxylates 3-polyprenyl-4-hydroxybenzoic acid **(7)**, has to hydroxylate 3-polyprenyl-2,4-dihydroxybenzoic acid in 2,4-diHB treated cells. (iv) The 4-HB analogs should be retained in the body unlike pABA and must be non-toxic, like vanillic acid which is licensed as a food additive (Gitzinger et al., 2012). However, other analogs may not be as innocuous, since control mice treated with 2,4-diHB gained less body weight than untreated mice (Wang et al., 2015) and high doses of 2,3,4-tri-HB were toxic in cell lines (Herebian et al., 2017).

## Conclusion

As demonstrated in yeast, mice, and human cell cultures, 4-HB analogs can bypass deficiencies in some steps of CoQ biosynthesis. 4-HB itself could be used to compensate for defects in the tyrosine to 4-HB pathway. These strategies are only possible thanks to a detailed molecular and genetic understanding of the CoQ biosynthetic pathway and efforts must continue to elucidate the steps that remain uncharacterized to date. In specific cases of primary CoQ deficiency, providing 4-HB analogs to reactivate the endogenous production of CoQ may represent a therapeutic alternative to CoQ_10_ supplementation. Further, investigations with animal models will establish whether this approach is realistic.

## Author contributions

The author confirms being the sole contributor of this work and approved it for publication.

### Conflict of interest statement

The author declares that the research was conducted in the absence of any commercial or financial relationships that could be construed as a potential conflict of interest.
